# Updates in gynecologic care for individuals with lynch syndrome

**DOI:** 10.3389/fonc.2023.1127683

**Published:** 2023-03-01

**Authors:** Kaylee A. Underkofler, Kari L. Ring

**Affiliations:** Department of Obstetrics and Gynecology, Division of Gynecologic Oncology, University of Virginia, Charlottesville, VA, United States

**Keywords:** lynch syndrome, endometrial cancer, ovarian cancer, genetics, gynecology, gynecologic oncology

## Abstract

Lynch syndrome is an autosomal dominant hereditary cancer syndrome caused by germline pathogenic variants (PVs) in DNA mismatch repair genes (*MLH1*, *MSH2*, *PMS2*, *MSH6*) or the *EPCAM* gene. It is estimated to affect 1 in 300 individuals and confers a lifetime risk of cancer of 10-90%, depending on the specific variant and type of cancer. Lynch syndrome is the most common cause of inherited colorectal cancer, but for women, endometrial cancer is more likely to be the sentinel cancer. There is also evidence that certain PVs causing Lynch syndrome confer an increased risk of ovarian cancer, while the risk of ovarian cancer in others is not well defined. Given this, it is essential for the practicing gynecologist and gynecologic oncologist to remain up to date on the latest techniques in identification and diagnosis of individuals with Lynch syndrome as well as evidence-based screening and risk reduction recommendations for those impacted. Furthermore, as the landscape of gynecologic cancer treatment shifts towards treatment based on molecular classification of tumors, knowledge of targeted therapies well-suited for mismatch repair deficient Lynch tumors will be crucial. The objective of this review is to highlight recent updates in the literature regarding identification and management of individuals with Lynch syndrome as it pertains to endometrial and ovarian cancers to allow gynecologic providers the opportunity to both prevent and identify Lynch-associated cancers earlier, thereby reducing the morbidity and mortality of the syndrome.

## 1 Introduction

Lynch syndrome, first recognized in 1895, is a well-defined hereditary cancer syndrome that affects approximately 1 in 300 individuals in the general population (1, 2). It is an autosomal dominant condition that is caused by pathogenic variants (PVs) in DNA mismatch repair (MMR) genes (*MLH1*, *MSH2*, *PMS2*, *MSH6*) or the *EPCAM* gene, which causes upstream promoter hypermethylation of *MSH2*. The lifetime risk of developing cancer among those with Lynch syndrome is highly variable, ranging from 10-90%, and is now understood to be related to the specific pathogenic variant (PV) causing the disorder in an individual or family (3–5). For example, those with a PV in *MLH1* have a 71-90% lifetime risk of any Lynch cancer and a 35-90% lifetime risk of colorectal cancer whereas those with a PV in *PMS2* have a 34-52% lifetime risk of any Lynch cancer and a 12-52% risk of colorectal cancer.

While it was first defined for its association with colon cancer, Lynch syndrome also increases the lifetime risk of cancer of the endometrium, ovary, stomach, small bowel, pancreas, brain, and genitourinary system (6). Endometrial cancer is the most common extracolonic cancer and is often the sentinel malignancy in women (7). Lynch syndrome is thought to cause 3% of colon cancers, and it is also thought to cause 3% of endometrial cancers (8–11). Endometrial cancer is most strongly associated with PVs in *MLH1*, *MSH2*, and *MSH6*, which confer a lifetime risk of endometrial cancer of 34-54%, 21-57%, and 16-49%, respectively (3–5, 12). Ovarian cancer is also associated with Lynch syndrome, specifically with PVs in *MSH2* and *MLH1*, which confer an 8-38% and 4-20% lifetime risk of ovarian cancer, respectively. In contrast, updated evidence in *MSH6* and *PMS2* carriers does not show a definitive increased lifetime risk of ovarian cancer, which is different from broader non-variant risk estimates utilized in the past (**Table 1**). Lynch associated cancers are also diagnosed at an earlier age than their general population counterparts (9). The mean age at the time of endometrial cancer diagnosis in those with Lynch syndrome is 47-55 years compared to age 60 in those without Lynch syndrome, and this same pattern is observed with ovarian cancer (13).

**Table 1:** Estimated lifetime risks of gynecologic cancers in Lynch syndrome.

Variant	Population EC Risk 3.1%		Population OC Risk 1.3%	
	EC Risk	EC Average Age	OC Risk	OC Average Age
*MLH1*	34-54%	49	4-20%	46
*MSH2/EPCAM*	21-57%	47-48	8-38%	43
*MSH6*	16-49%	53-55	≤1-13%	46
*PMS2*	13-26%	49-50	1.3-3%	51-59

EC, endometrial cancer; OC, ovarian cancer (12).

These facts highlight the importance of women’s health provider familiarity with Lynch syndrome. Methods of successful endometrial cancer risk reduction among women with Lynch Syndrome have been identified, such as total hysterectomy (14). Therefore, this condition should be in the forefront of the gynecologist’s and gynecologic oncologist’s mind when seeing patients with endometrial or ovarian cancer or a suggestive personal or family history to assist with preventive efforts. Women’s health providers should be prepared to discuss the diagnosis, lifetime risk of malignancy, as well as recommended screening and risk reduction methods. Gynecologic oncologists can take this discussion a step further with recent evidence supporting targeted treatments for MMR deficient tumors associated with Lynch syndrome. The objective of this review is to highlight recent updates in the literature regarding these topics of identification and management of individuals with Lynch syndrome as it pertains to endometrial and ovarian cancers. This may allow gynecologic providers the opportunity to both prevent and identify Lynch-associated cancers earlier, thereby reducing the morbidity and mortality of the syndrome.

## 2 Identification of individuals with lynch syndrome

The first step in reducing morbidity and mortality of Lynch syndrome on a population level is to identify which individuals may be at risk and qualify for germline genetic testing. This unfortunately is also one of the most challenging steps. Lynch syndrome is suspected to be underdiagnosed in the general population (15). Many screening tools have been created over the years to improve carrier identification, including those based on family history such as the Amsterdam Criteria, clinical prediction models, as well as screening on colon, endometrial, and ovarian tumors. The sensitivity and specificity of these methods, as well as their cost, vary greatly, and importantly, providers must have a high pretest suspicion of Lynch syndrome to employ them effectively. Once an individual is determined to be high-risk, diagnostic testing in the form of germline genetic tests for MMR and *EPCAM* PVs is more straightforward.

### 2.1 Screening methods to identify individuals at risk for lynch syndrome

As previously stated, there are many tools in existence to identify who should have genetic testing to establish a diagnosis of Lynch Syndrome. Unfortunately, many providers are unaware of these tools and if they do screen patients for hereditary cancer syndromes, they do so based on the classic findings of early age of diagnosis of cancers or multiple Lynch associated cancers in family members over use of validated screening tools (16, 17).

Use of family-history based criteria are the earliest standardized methods proposed for who should be tested for Lynch syndrome. Use of the Amsterdam II Criteria is one of these methods, and recommends testing an individual for Lynch syndrome when they meet all of the following criteria: 1) having 3 relatives with any Lynch-associated cancer with 1 being the first degree relative of the other 2, 2) there are 2 successive generations are affected, and 3) 1 is diagnosed before the age of 50 (18). The sensitivity and specificity of the Amsterdam II Criteria have since been determined to be 25-72% and 78-98%, respectively (19, 20). The low sensitivity is certainly a weakness of this screening method, though a benefit is that it does not require an individual to already be diagnosed with a cancer prior to screening, and considers Lynch-associated cancers other than colorectal cancer, which is important for those approaching screening from a women’s health perspective. The Amsterdam II Criteria were followed by the Bethesda Criteria and Revised Bethesda Criteria, a set of guidelines based on personal and family history for when patients with colorectal cancer should have their tumors tested for microsatellite instability (MSI), a pathologic hallmark of Lynch-associated cancers (21). The sensitivity and specificity of the Revised Bethesda Criteria were determined to be 50-94% and 25-75% respectively (19, 20). In addition to a lower specificity, from a gynecologic perspective, this screening method is limited by the fact that it requires a colorectal cancer diagnosis and does not take into account endometrial and ovarian malignancies, though the American College of Obstetricians and Gynecologists (ACOG) has made modifications to extrapolate the criteria to gynecologic cancers (22). Furthermore, it is not able to identify individuals with Lynch syndrome prior to a cancer diagnosis. The Society of Gynecologic Oncologists (SGO) also developed guidelines in 2007 that placed individuals within two risk categories (20-25% risk and 5-10% risk) of having Lynch syndrome that were based on the Amsterdam criteria and Bethesda criteria (23). However, follow-up studies show a relatively low sensitivity of SGO criteria consistent with studies of Amsterdam and Bethesda criteria (24, 25).

Clinical prediction models were developed to improve the detection of individuals with Lynch syndrome compared to methods based primarily on family history such as the Amsterdam II Criteria and Revised Bethesda Criteria. Their strength is that they screen for Lynch syndrome prior to a person being diagnosed with cancer. Several models have been created, such as MMRpredict, MMRpro, and PREMM5 (26–28). Each model is somewhat different based on variables they take into account, including characteristics such as age, sex, age at diagnosis of cancer, family history of cancer with family ages of diagnosis, and testing results, if available. However, each is similar in that they are designed to quantify the likelihood a person has a PV in an MMR gene, guiding counseling for the decision to pursue genetic testing. The advantage of these models is that they are simple, validated tools for providers to employ in cancer unaffected individuals that may improve upon the screening test characteristics of the Amsterdam II Criteria and the Revised Bethesda Criteria, though comparative studies are few and conflicting (26, 29, 30). However, it is important to note that not all models quantify risk for PVs in all Lynch-associated genes. Importantly, it should be noted that MMRpredict is validated for patients with colorectal cancer rather than endometrial or ovarian cancers, while MMRpro considers endometrial cancer and PREMM5 considers both endometrial cancer and other Lynch-associated cancers including ovarian cancer (26–28). Gynecologic providers must be aware of this when selecting a clinical prediction model if this is the screening method they choose to utilize.

The current standard of care in screening for Lynch syndrome in those who are affected by cancer is tumor-based testing of patients for loss of expression of MMR proteins with immunohistochemical staining (IHC) (31). IHC staining detects the presence of MMR proteins, and staining is lost when there is a loss or defect in an MMR gene as is seen in Lynch syndrome. This is generally an indication for germline MMR gene testing for diagnosis of Lynch syndrome, though there are additional steps such as *MLH1* hypermethylation testing depending on the pattern of loss of expression visualized to determine whether the loss of expression is sporadic or a result of a germline PV. An adjunct or lesser alternative to IHC staining is tumor-based MSI testing (11). MSI testing is traditionally performed using polymerase chain reaction (PCR) to identify expansion or contraction of repetitive DNA sequences within the tumor that are prone to error, and is recommended when IHC results are equivocal. Tumors that show a certain degree of this expansion or contraction are determined to be MSI-high (MSI-H). Most Lynch tumors are MSI-H, but only about 16% of MSI-H tumors are associated with Lynch syndrome (32). MSI-H tumors are, however, an indication for Lynch genetic testing if identified, regardless of type of malignancy. Tumor testing as a screening method offers the greatest sensitivity and specificity of the methods described, but unfortunately requires a cancer diagnosis for screening to be completed, thus limits primary prevention of cancer in those with Lynch syndrome, though it does offer options for prevention of metachronous malignancies.

When it comes to tumor testing as a method of screening for Lynch syndrome, a key question is knowing which tumors to test. The Revised Bethesda Criteria offer one solution to this question, though may miss 12-30% of Lynch-associated tumors and would need modification and ideally validation for patients with endometrial or ovarian cancer (8). Universal screening of tumors allows the greatest detection of Lynch syndrome, but whether or not it is truly cost-effective remains in question. Studies in colorectal cancer populations support universal colorectal tumor testing as reasonably cost-effective (33). A study in the United Kingdom also found universal IHC staining of endometrial tumors to be cost-effective (34). However, a cost-effectiveness study on a variety of testing criteria in women with endometrial cancer in the United States calculated an incremental cost-effectiveness ratio (ICER) of $648,494 per life year gained for universal endometrial cancer tumor testing, which was significantly greater than $9,126 per year of life gained for the recommended strategy of testing the tumors of all women endometrial cancer with at least 1 first degree relative with Lynch-associated cancer diagnosed at any age (35). Regardless of the cost, because of enhanced detection of Lynch syndrome and prognostic implications of certain molecular subtypes of endometrial carcinomas, universal tumor testing is now recommended by several professional societies, including the National Comprehensive Cancer Network (NCCN), Society of Gynecologic Oncology (SGO), ACOG, and European Society for Medical Oncology (ESMO) (22, 36–38) Whether or not universal endometrial tumor testing as a screening method for Lynch syndrome is performed and the extent of testing may be institution-dependent at this time due to cost and pathology expertise. This poses an issue for equitable care and hopefully further study and technology advances can standardize screening. As for ovarian cancer, NCCN guidelines recommend germline and tumor testing for all patients diagnosed to not only evaluate for Lynch syndrome, but also to evaluate for *BRCA* mutations and other molecular features that may influence treatment decisions (39). Current guidance from SGO on identifying patients with an increased likelihood of Lynch syndrome takes into account family history as well as molecular based tumor screening techniques (**Table 2**) (37).

**Table 2:** Patients at increased risk of LS for whom genetic assessment is recommended (modified from SGO statement on risk assessment for inherited gynecologic cancer predispositions).

Patients with EC or CRC with evidence of MSI or loss of DNA MMR protein (MLH1, MSH2, MSH6, PMS2) on IHC.
Patients with FDR affected with EC or CRC diagnosed <60 years or identified to be at risk for LS by systematic clinical screen that incorporated focused personal and medical history.
Patients with FDR or SDR with a known pathogenic variant in a MMR gene.

EC, endometrial cancer; CRC, colorectal cancer; MSI, microsatellite instability; MMR, mismatch repair; FDR, first degree relative; LS, Lynch syndrome; SDR, second degree relative; IHC, immunohistochemistry (37).

Despite gradual improvements in the detection of individuals at risk for Lynch syndrome, there continues to be a significant number who remain undiagnosed, and development of novel screening strategies or technology to improve access to screening should be a priority. One suggested solution includes use of remote genetic counseling to better identify high-risk individuals who may not have access to in-person genetic counseling, which has been shown to produce similar levels of patient knowledge and satisfaction and reduces costs, but may result in lower counseling and testing completion rates compared to in-person counseling (40, 41). Additionally, genetic counseling itself requires a provider referral, which adds a step in the screening process and therefore adds a barrier. Genetic counseling by providers other than genetic counselors has been explored to remove this barrier, though this is dependent on provider acceptability of and comfortability with performing their own genetic counseling. One recent study evaluated the feasibility of gynecologist led Lynch syndrome counseling and testing, rather than sending a patient to a genetic counselor prior to testing, and results were favorable in terms of acceptability by women being tested and uptake of testing upon counseling (42). Another proposed solution to the genetic counselor barrier suggests use of health information technology in the form of chatbots with which individuals can directly interact (43). These chatbots use an individual’s input and standardized risk assessment tools to produce a risk estimate for hereditary cancer syndromes. They can then facilitate genetic testing, counsel regarding results, and assist with cascade testing virtually. One study evaluating use of a chatbot revealed patient knowledge and genetic testing completion rates similar to those achieved through genetic counseling, but low rates of individuals initiating interaction with the chatbot and low provider interest or comfort in results follow-up (44). Studies evaluating patient and provider acceptability of chatbots and process implementation are ongoing. Population-based genetic testing for germline PVs has also been proposed given improvements in DNA sequencing and lower costs (45). Indeed, this method identifies many individuals with hereditary cancer syndromes who would not otherwise meet high-risk criteria, but is not cost-effective at this time, and the stress of finding variants of unknown significance (VUS) may adversely affect some individuals.

### 2.2 Diagnosis of lynch syndrome

Once a person has been screened as high-risk for Lynch syndrome using one of the methods above, the definitive diagnosis can be established through germline testing. This can be done through several methods: multigene panel testing, targeted MMR gene testing, or single gene testing. The National Comprehensive Cancer Network (NCCN) recommends single gene testing if there is a known PV within a person’s family and if they are clinically low-risk for other PVs (46). Multigene panel testing, in which several cancer predisposition PVs are sequenced, is recommended for those at high-risk for Lynch syndrome but without a known PV in the family due to the possibility of another hereditary cancer syndrome placing the individual in the high-risk category. MMR PV testing may be best for those with a specific IHC pattern after tumor testing. Multiple professional societies, including ACOG and NCCN, agree that it is best practice to involve cancer genetics experts, such as genetic counselors, whenever genetic testing may be performed. However, given a national shortage of these skilled professionals, this may not be a resource for all, and presents an area for improved access for equitable high-risk care (22, 46).

Interpretation of genetic testing results is key to counseling(**Table 3**) (47). VUS are changes or alterations in the genetic code for which the downstream protein function is unknown and are sometimes the most clinically challenging result to contextualize for patients. As there is increased utilization of multigene panels in broader populations, more VUSs will be identified (48). Approximately 80-90% of VUSs will subsequently be reclassified as benign polymorphisms and should be treated as clinically negative (49). In addition, an increasing number of individuals present to care having completed direct to consumer testing (DTC), where these individuals interact directly with testing companies. There is a wide range of DTC companies with different testing methodology and interpretation of results. Currently, any PV identified on DTC should be verified through a clinical lab (47). Lastly, there are families that meet Amsterdam criteria and a germline PV is not identified in the family. These individuals may be followed as having clinical Lynch syndrome, however, this should be done in consultation with a high-risk expert.

**Table 3:** Interpretation of germline genetic testing results (47).

Result	Description
True Positive	Individual is a carrier of an alteration in a known cancer-predisposing gene
True Negative	Individual is not a carrier of a known cancer-predisposing gene that has been positively identified in another family member
Indeterminate	Individual is not a carrier of a known cancer-predisposing gene and carrier status of other family members is also negative or unknown
Variant of Unknown Significance	Individual is a carrier of gene alteration that currently has no known significance

Once an individual is diagnosed with Lynch syndrome, they should be counseled regarding both their personal risk of cancer and also their family’s potential risk. This should include a conversation regarding cascade testing, which involves genetic testing of a known carrier’s relatives to determine whether these family members are affected, and thus also at increased risk. Cascade testing is recommended to begin with first-degree relatives, as these individuals have a 50% chance of having the same Lynch syndrome PV, and if positive, expand cascade testing to their first-degree relatives (50). Generally, these individuals need only be tested for the known PV that has been identified in their family rather than testing for all PVs associated with Lynch syndrome. Cascade testing enhances the ability to identify more carriers with Lynch syndrome that otherwise might not be screened, and improves the cost-effectiveness of universal tumor testing (51).

Another consideration to offer individuals interested in reproduction upon diagnosis of Lynch syndrome is referral to a reproductive endocrinology and infertility (REI) specialist to discuss preimplantation genetic testing (PGT). This diagnostic test is used in conjunction with *in vitro* fertilization (IVF), and involves testing an embryo in the lab for a specific PV to reduce the risk of passing this PV on to future children. For Lynch syndrome, this would mean testing the embryos of a Lynch syndrome carrier and their partner for the specific MMR PV the carrier is known to have. Identifying which embryos carry this PV allows the REI specialist to inform the parents undergoing IVF and selectively transfer embryos that are not carriers, thus preventing a future child from being affected by Lynch syndrome. It is important to address the timing of IVF if the Lynch syndrome carrier is female, as it may be affected by the recommended timing of risk-reducing hysterectomy to prevent endometrial cancer or risk-reducing oophorectomy to prevent ovarian cancer, as is discussed in the next section (52).

## 3 Gynecologic cancer screening and risk reduction in individuals with lynch syndrome

Once a diagnosis of Lynch syndrome is established, it is recommended to begin the process of screening for early development of Lynch-associated cancers and in some instances undergo risk-reducing procedures or initiate chemoprevention under the care of physicians with expertise in the management of high-risk carriers. While screening and risk reduction methods exist for other Lynch-associated cancers, colonoscopy screening for colorectal cancer being at the forefront, this review will focus on those measures targeted towards the screening and prevention of endometrial and ovarian cancers in those with Lynch syndrome.

### 3.1 Endometrial cancer

Multiple approaches to screening for endometrial cancer in asymptomatic women diagnosed with Lynch syndrome have been proposed, including endometrial biopsy and transvaginal ultrasound (TVUS). Importantly, none of these methods have been shown to reduce the morbidity and mortality of women with Lynch syndrome (53–56). This is likely due to the fact that the majority of endometrial cancers are already diagnosed with early stage disease and any screening intervention will not improve dramatically on the early stage of diagnosis overall for endometrial cancer. Despite a lack of proven efficacy, and given the low risk of screening tests and high risk of endometrial cancer in this population, multiple professional societies including ACOG and NCCN agree that endometrial biopsy every 1-2 years starting between the ages of 30 and 35 can be considered in women diagnosed with Lynch syndrome (22, 46). Many experts go on to recommend starting screening with endometrial biopsy 10 years before the earliest Lynch-associated cancer diagnosis in the family and to continue endometrial biopsies until the time of hysterectomy. Endometrial biopsy is the test of choice due to its excellent sensitivity of 91-99.6% and specificity of 98% for endometrial cancer and hyperplasia, as well as evidence that it enhances detection of endometrial cancer and hyperplasia compared to TVUS alone (54, 57). It is, however, an invasive and uncomfortable test, which may impact acceptability to patients. There is prospective evidence from patient reported outcomes that performing endometrial biopsy at the time of colonoscopy decreased pain associated with the biopsy (58). While this was shown to be feasible in the setting of a study, whether this is feasible in practice depends on many factors, most notably where colonoscopies are performed within individual practices.

TVUS is less invasive than endometrial biopsy, however, it offers lower detection rates and it is not recommended in premenopausal females since endometrial thickness varies greatly throughout a menstrual cycle (46, 54). A few studies have investigated the combination of endometrial biopsy and TVUS, which shows promise in increasing detection, but again, does not offer definitive morbidity or mortality benefit at this time (55, 59).

Additional methods of screening for endometrial cancer have been proposed and show potential, but currently lack sufficient evidence supporting efficacy required of a suitable screening test in a clinical setting. The use of pap smears and tampon-based intravaginal sampling to detect cancerous endometrial cells, shed tumor DNA, or biomarkers are a few of these methods (60–63). One such study evaluating 18 genes in fluid collected *via* pap smear for endometrial and ovarian cancer patients in comparison to cancer-free controls found a specificity of 99%, though it was limited by modest sensitivity (78% for endometrial cancer, 33% for ovarian cancer) (63). Analysis of urinary samples for potential endometrial cancer biomarkers, such as estrogen metabolites, has also been proposed by several studies and is interesting as a simple, non-invasive form of screening, but to date none of the biomarkers suggested have been validated (64–66). Each of these will be ideas to watch over the next several years.

For women diagnosed with Lynch syndrome who present with symptoms, including but not limited to abnormal bleeding, pelvic mass or pain, abnormal discharge, or weight loss, women’s health providers should have high suspicion for endometrial cancer and investigation in the form of endometrial biopsy and/or transvaginal ultrasound should be performed.

Risk reduction of endometrial cancer is another aspect of management of which providers and women with Lynch syndrome need to be aware (**Table 4**). The most invasive method, and that with the greatest evidence for prevention of endometrial cancer in this population, is risk-reducing hysterectomy and bilateral salpingectomy with or without oophorectomy. The largest study comparing outcomes between women with Lynch syndrome who underwent risk-reducing surgery and those who did not found that 0% of patients who underwent hysterectomy were diagnosed with endometrial cancer after 13 years of follow-up compared to 33% of those who did not have a hysterectomy (14). The timing of this intervention is controversial given lack of strong evidence dictating a specific age, though desire for fertility, age at cancer diagnosis in Lynch-affected family members, diagnosis of other cancers, and even specific PV should all be considered (46). Given a 4-fold increase in endometrial cancer risk from the age of 40 to the age of 50, many professional societies, including ACOG and SGO, recommend risk-reducing hysterectomy by the age of 40-45 (4, 22). Surgery before age 40 can also be considered if a woman has completed child-bearing, and indeed, the American Society for Clinical Oncology (ASCO) recommends this (67). A cost-effectiveness analysis comparing multiple ages for surgery found that annual screening until hysterectomy at age 40 was most effective at preventing endometrial cancer, but risk-reducing surgery at age 40 without screening was the most cost-effective given the substantial cost of screening and hormone replacement therapy for surgical menopause when surgery was performed at age 30 (68). Despite these results, expert consensus is to continue screening for endometrial cancer until hysterectomy is performed, at which point it can be discontinued. For those desiring fertility, referral to a reproductive endocrinologist should be considered prior to surgery, especially if they are approaching advanced maternal age. If colorectal cancer is diagnosed prior to risk-reducing gynecologic surgery, a joint procedure with colorectal surgery can be planned and this can also dictate timing of surgery. Regardless of timing, colonoscopy and endometrial biopsy should be up to date prior to risk-reducing surgery to rule out occult malignancies and be sure the appropriate procedure is being planned.

**Table 4:** Expert screening and risk reduction recommendations for gynecologic cancers in Lynch syndrome, modified from NCCN Guidelines (Version 2.2022: Lynch Syndrome) (12).

Variant	EC Screening and Risk Reduction	OC Screening and Risk Reduction
*MLH1*	EBx every 1-2 years starting at 30-35 can be considered Consider RR agents Hysterectomy as RR option can be considered, timing based on patient factors	TVUS and CA125 may be considered at clinician’s discretion Consider RR agents BSO as a RR option should be individualized
*MSH2/EPCAM*	EBx every 1-2 years starting at 30-35 can be considered Consider RR agents Hysterectomy as RR option can be considered, timing based on patient factors	TVUS and CA125 may be considered at clinician’s discretion Consider RR agents BSO as a RR option should be individualized
*MSH6*	EBx every 1-2 years starting at 30-35 can be considered Consider RR agents Hysterectomy as RR option can be considered, timing based on patient factors	TVUS and CA125 may be considered at clinician’s discretion Consider RR agents Insufficient evidence for specific recommendation, BSO as a RR option should be individualized
*PMS2*	EBx every 1-2 years starting at 30-35 can be considered Consider RR agents *PMS2* carriers appear to be at only a modestly increased risk of EC: Hysterectomy as RR option can be considered, timing based on patient factors	TVUS and CA125 may be considered at clinician’s discretion Consider RR agents Insufficient evidence for specific recommendation, *PMS2* carriers appear to be at no greater than average risk, BSO as a RR option should be individualized in consultation with gynecologist with expertise in LS

Note that these are the recommendations of NCCN, and that recommendations may vary depending on the professional organization. EC, endometrial cancer; OC, ovarian cancer; EBx, endometrial biopsy; RR, risk reducing; BSO, bilateral salpingo-oophorectomy; TVUS, transvaginal ultrasound.

Lifestyle and medical chemoprevention options should also be discussed with individuals with Lynch syndrome. In accordance with general population recommendations, those with Lynch syndrome should be counselled to maintain or attain a normal body weight given the well-defined association of obesity with the development of endometrial cancer. They should also be counselled to engage in 30 minutes of exercise daily or 150-300 minutes of moderate intensity exercise weekly exercise per American Cancer Society (ACS) guidelines (69).

As for medical chemoprevention, there is evidence that daily aspirin may be associated with a reduction in all Lynch-associated cancer diagnoses in those with Lynch syndrome (70). The CAPP2 trial is a multinational randomized controlled trial examining differences in colorectal and all Lynch cancer diagnoses in those with Lynch syndrome based on use of daily aspirin. Individuals were randomized to receive either placebo or 600mg of aspirin daily. After an average of 10 years of follow-up, colorectal cancer and all Lynch cancer diagnoses were found to be significantly lower in those taking daily aspirin, though there was no difference noted for all non-colorectal Lynch cancer diagnoses. While the benefit for endometrial or ovarian cancers is unclear, there is benefit for colorectal cancer and thus women with Lynch syndrome can be offered daily aspirin for their comprehensive care if there are no contraindications.

Hormonal therapies, including combined oral contraceptive pills (OCPS), oral progestins, or progesterone containing intrauterine devices (IUDs), are an alternative form of risk-reduction for those with Lynch syndrome prior to completion of childbearing and risk-reducing hysterectomy. Data for endometrial cancer prevention with hormonal therapies in the Lynch population specifically are limited and the majority of evidence is extrapolated from studies in the general population (71–73). Population based evidence shows that OCP use for 5 years decreases endometrial cancer risk by 50-70%, with increased protection with longer duration of treatment (74). Similarly, retrospective evaluation showed a 61% risk reduction in endometrial cancer among women with Lynch syndrome who used hormonal contraception in the form of combined or progestin only pill, the implant, or the injection for at least one year (75). A small randomized controlled trial examined the effect of Depo-Provera versus progestin-only oral contraceptives in a population of women with Lynch syndrome, which revealed a decrease in endometrial proliferation in both groups, but was not able to compare endometrial cancer rates between the groups (76). Progesterone containing IUDs, which are now utilized to treat endometrial intraepithelial neoplasia and low grade endometrial cancers, are also associated with an approximate 50% decreased risk of endometrial cancer in the general population and this decreased risk persists for 5 year following discontinuation (77–79).

### 3.2 Ovarian cancer

As with endometrial cancer, multiple screening methods for the early detection of ovarian cancer have been proposed for asymptomatic women with Lynch syndrome, though no evidence exists supporting an improvement in morbidity or mortality for any method (55, 56). Annual transvaginal ultrasound is one method that has been studied, but has relatively poor sensitivity and specificity for ovarian cancer and thus can be considered, but is not formally recommended by major professional gynecologic or oncology professional organizations such as ACOG, SGO, or NCCN (22, 46, 80). The same is true for the measurement of CA 125. Importantly, there are no studies on these screening methods in the Lynch syndrome population specifically, they are only available from the general population or among those with BRCA mutations (81–84). This is problematic because Lynch-associated ovarian cancer is different from BRCA-associated ovarian cancer. Lynch associated ovarian cancers tend to be mostly endometrioid and have a more favorable prognosis than the aggressive serous ovarian cancers associated with BRCA mutations (85). They also derive from different molecular pathways. Thus, combining these two very different types of ovarian cancers under one umbrella based on evidence availability, or lack thereof, should be done with great caution. Certainly, more studies in a Lynch population are needed.

Any female with Lynch syndrome presenting with bloating, a palpable mass, abdominal pain, weight gain or loss, early satiety, or other concerning symptoms should undergo imaging to determine if ovarian cancer is present.

Risk-reducing surgery for the prevention of ovarian cancer in those with Lynch syndrome is currently one of the most difficult clinical questions to consider in high-risk care for the individual as differential lifetime risks of ovarian cancer for specific Lynch variants have been better outlined in recent years (**Table 4**). Most data evaluating risk reduction for ovarian cancer include bilateral salpingo-oophorectomy (BSO) with tubes and ovaries removed at the same time and includes data for all variants in aggregate, rather than for individual variants. This is critical to understand for counselling, especially those with PV in *MSH6* and *PMS2*, where there is no strong recommendation for oophorectomy based on current available evidence. It should be noted throughout the discussion that regardless of the recommendation for bilateral oophorectomy, bilateral salpingectomy is recommended at the time of risk reducing hysterectomy (86, 87).

NCCN currently recommends for *MLH1* and *MSH2/EPCAM* carriers that the decision to have a BSO as a risk-reducing option should be individualized and timing should be based on completion of childbearing, menopausal status, medical comorbidities, family history, and specific variant. Differently, NCCN states that for *MSH6* carriers, insufficient evidence exists to make a specific recommendation for BSO and that the decision should be individualized. They are even more detailed in their recommendation for *PMS2* carriers and state that *PMS2* carriers appear to be at no greater risk of ovarian cancer and that individuals may reasonably elect not to have an oophorectomy (12).

In a study evaluating BSO compared to no BSO for the prevention of ovarian cancer in 223 individuals with Lynch syndrome, no one who underwent BSO was diagnosed with ovarian cancer, while 5% who did not undergo BSO were ultimately diagnosed with ovarian cancer, supporting BSO as a reasonable method of risk reduction (14). Ovarian cancer risk in those with Lynch syndrome triples from the age of 40 to the age of 50 depending on the PV, therefore timing recommendations of surgery before age 45 are the similar to endometrial cancer (4). Hormone replacement therapy (HRT) is generally considered safe for the treatment of surgical menopause after BSO in premenopausal women, though has not been directly studied in a Lynch population. Given that hysterectomy is also recommended for risk reduction, women benefit from needing estrogen replacement therapy (ERT) alone. Expert opinion is for consideration of HRT for women with Lynch Syndrome who undergo premenopausal BSO (46). Interestingly, there is evidence of a protective effect of HRT against the development of colorectal cancer in the general population, and this may be helpful for women with Lynch syndrome who have an increased risk of colorectal cancer, though further study in this specific population would be needed (88).

For the individual, discussion of the risks and benefits of oophorectomy is paramount in the Lynch population and should include a gynecologic provider experienced in high-risk care. For most, the decision is whether to proceed with oophorectomy at the time of hysterectomy and bilateral salpingectomy. Several factors should be taken into account on top of completion of childbearing, most notably age, family history of ovarian cancer and age of diagnosis, as well as other medical and surgical co-morbidities. Regardless of the individual variant, if an individual is ready to proceed with risk reducing hysterectomy at 35, a well-documented and thorough discussion of oophorectomy is necessary, most notably including the risk of early surgical menopause, including increased risk of osteoporosis, cardiovascular disease, and effects on cognitive as well as sexual function. In addition, there is mounting evidence that the majority of high grade serous ovarian cancers originate in the distal fallopian tube. Opportunistic salpingectomy is associated with a 42-64% reduction in the risk of ovarian cancer in epidemiologic studies (89–91). There are ongoing studies in the BRCA population for this, but the degree to which salpingectomy decreases the risk of Lynch associated ovarian cancers specifically is largely unknown, where the incidence of non-serous ovarian cancers are higher than in the BRCA population (92). Delayed oophorectomy is an option for those who wish to defer menopause with appropriate counselling, though this would require a second surgery if hysterectomy is done earlier and surgery thus may be more complicated (93).

Ovarian cancer chemoprevention with combined estrogen and progestin oral contraceptive pills (COCPs) is an option for women with Lynch syndrome, though again, there are no studies in the Lynch syndrome population. Studies in the general or BRCA populations do suggest a benefit in ovarian cancer prevention with the use of COCPs, but again, Lynch-associated ovarian cancer is fundamentally different than BRCA-associated ovarian cancer, thus it is not clear whether a true benefit exists in the Lynch syndrome population (46, 94–97). Premenopausal females with Lynch syndrome who have not completed childbearing and thus have not yet undergone risk-reducing hysterectomy and BSO can have a risk/benefit discussion with their provider to determine if chemoprevention with COCPs is the right choice for them.

An exciting intervention that may be on the horizon for cancer prevention in carriers of Lynch syndrome is that of cancer vaccines. These investigational vaccines are developed against neoantigens produced by frameshift mutations in those with MSI-H tumors and Lynch syndrome (98). There are currently multiple registered clinical trials investigating the development of vaccines to prevent cancer in those with Lynch syndrome specifically (99).

## 4 Treatment of gynecologic cancer in patients with lynch syndrome

All treatment options that are available to those with sporadic endometrial and ovarian cancers are also available to those with Lynch-associated endometrial and ovarian cancers, and prior to molecular analysis of tumors, treatment recommendations were the same regardless of Lynch status. Since the relatively recent discovery that MMR deficient and MSI-H tumors, the key characteristics of Lynch tumors, may be more susceptible to immunotherapy that functions *via* PD-1 blockade than tumors without these features, there is now evidence that patients with Lynch-associated tumors may benefit from alternative treatment plans (100). It is worthwile to note here that MSI-H tumors and MMR deficient tumors that arise sporadically may have a different prognosis than those that arise due to Lynch syndrome, and therefore applying research on treatments in all MMR-deficient tumors to Lynch syndrome must be done with caution, though studies in the Lynch syndrome population alone are limited (9).

Whether initial adjuvant therapy for endometrial cancer should be dictated by MMR deficiency is controversial based on available evidence. A retrospective study comparing outcomes between MMR deficient endometrial tumors and MMR proficient endometrial tumors following adjuvant therapy with either radiation or chemotherapy revealed a trend toward lower recurrence rates among patients with MMR deficiency, but on multivariate analysis, there was no association with progression-free or overall survival (101). However, a separate retrospective study found improved survival when patients with MMR-deficient endometrial cancer were treated with radiation therapy compared to those who were MMR-proficient (102). A third retrospective study found worse recurrence-free survival after vaginal brachytherapy in those with MMR-deficient endometrial cancer compared to those with MMR proficiency (103). When comparing chemoradiation to radiation therapy alone as adjuvant therapy for MMR deficient tumors of women in the PORTEC-3 population, no benefit was found with the addition of chemotherapy (104). Other studies are underway to investigate the influence of various adjuvant treatments on MMR deficient tumors, such at the MMRd-GREEN Trial under the RAINBO program, which is prospectively examining recurrence-free survival between patients with MMR deficient high-risk endometrial tumors randomized to receiving either radiation therapy alone or durvalumab, an immunotherapeutic, with radiation therapy (105). Furthermore, both PORTEC-4a and a clinical trial from China are currently investigating adjuvant therapies for early stage endometrial cancer based on either molecular classification (such as MMR-deficient) or traditional risk stratification (106, 107).

Regarding radiation therapy in those with Lynch-associated endometrial cancer, one should also consider the possibility of second primary malignancies after radiation treatment. While evidence is unavailable for an increased risk of second primaries attributable to radiation treatment for endometrial cancer specifically in a Lynch population, there is some evidence of increased risk for second primary malignancies after radiation for endometrial cancer in the general population (108). Other studies have found no increased risk of second primary malignancies attributable to radiation therapy for endometrial cancer (109). Insufficient evidence exists for formal recommendations, but if an association exists between radiation therapy for endometrial cancer and second primary malignancies, those at increased risk of second primary malignancies in the first place such as those with Lynch syndrome may need to be approached more cautiously with radiation therapy, and at the very least continue close surveillance with regular colonoscopies. Close communication between a patient’s gastroenterology and oncology teams is warranted in this situation.

Evidence regarding initial adjuvant therapy in MMR-deficient ovarian cancers is also conflicting. One retrospective study found similar survival rates between MMR-deficient and MMR-proficient ovarian cancers, thus recommended treating them similarly (13). Some studies report improved survival in MMR-deficient cases compared to MMR-proficient cases that could be considered when deciding on therapy, but not all took into account a higher likelihood of endometrioid histology associated with MMR deficiency, which is a significant prognostic factor (110–112). Another study reported improved survival in patients with high expression of MMR genes who were treated with platinum-based chemotherapy, supporting *in vitro* studies that called into question whether platinum resistance is associated with MMR deficiency (110, 113). At this time, given conflicting and limited evidence, there are no societal recommendations regarding MMR deficiency and initial adjuvant treatment in either endometrial or ovarian cancer. Further study is needed.

While studies are inconclusive regarding MMR deficiency and its influence on upfront therapy, recent evidence in favor of immunotherapy for recurrent or progressive MMR-deficient endometrial and ovarian cancers has emerged. The theory behind immunotherapy is to utilize the body’s own immune system to attack tumor cells, and this branch of treatment has been shown to be effective in multiple types of cancer. In fact, pembrolizumab, a monoclonal antibody that inhibits T-cell apoptosis by blocking the PD-1 receptor on these immune cells, is FDA approved for all non-colorectal, MSI-H and MMR deficient tumors, regardless of tumor site, and it the first therapy to receive accelerated approval for a tumor-agnostic indication (114). In gynecologic cancer specifically, pembrolizumab is the immunotherapeutic best studied and supported (100, 115). The largest trial on this topic presently is the Keynote 158 study, which published results of a phase II randomized controlled trial evaluating the efficacy of pembrolizumab in the treatment of non-colorectal, MSI-H and MMR-deficient tumors (116). The study included 49 cases of endometrial cancer and 15 cases of ovarian cancer. Results revealed a 34.3% objective response rate, supporting pembrolizumab as a treatment option in this population. NCCN now recommends pembrolizumab for the treatment of MSI-H and MMR deficient endometrial and ovarian tumors that fail to respond adequately to first-line therapy and this recommendation should be discussed with patients with Lynch syndrome (38, 39).. More clinical trials are underway evaluating immunotherapy among women with MMR-deficient advanced or recurrent endometrial tumors, such as the KEYNOTE-C93 trial investigating pembrolizumab versus platinum-doublet chemotherapy and the DOMENICA trial investigating dostarlimab, another immunotherapeutic, versus platinum-doublet chemotherapy (117, 118).

Whether immunotherapy will be of use for upfront treatment of Lynch-associated endometrial or ovarian cancers is under investigation. Not only will the MMRd-GREEN Trial shed light on this, there is also the IMHOTEP trial, which is currently underway investigating pembrolizumab as neoadjuvant therapy prior to surgical resection of MSI-H and MMR-deficient tumors of multiple sites (119). The use of immunotherapy in the neoadjuvant setting for MSI-H and MMR-deficient colorectal cancer has been reported with promising results in several case studies (120–122), and multiple clinical trials studying neoadjuvant immunotherapy are underway in this population. It will be exciting to monitor progress in this field over the next few years to determine whether neoadjuvant immunotherapy can have a similar impact on MMR-deficient endometrial and ovarian cancers.

## 5 Conclusion

While much has been discovered about our understanding of cancer risk and our ability to reduce risk for those with Lynch syndrome, there remains a great deal to be discovered to diminish its associated morbidity and mortality. Improvements in technology are needed to increase identification of individuals at high risk for Lynch syndrome, not only by utilizing high quality screening tests, but also for increased patient access to these tools and for reduction in costs to allow more universal testing. The same is true for screening for Lynch-associated cancers in patients diagnosed with Lynch syndrome, especially given that endometrial cancer and ovarian cancer screening has not yet been shown to have a mortality benefit. Identifying barriers and improving access to risk reduction measures is another future direction in the study of Lynch syndrome, and perhaps the greatest frontier is determining whether Lynch-associated endometrial and ovarian tumors should be treated differently than sporadic endometrial and ovarian tumors. Dedication to these efforts will bring about the implementation of important practice changes and hopefully afford us the mortality benefit in the management of Lynch-associated endometrial and ovarian cancers we have been seeking.

## Author contributions

KU was involved in concept development, writing, and editing. KR was involved in concept development, writing, and editing. All authors contributed to the article and approved the submitted version.
